# Terahertz Spectroscopic Insight into the Hydrogelation of Copper Ion-Coordinated Poly(vinyl alcohol)

**DOI:** 10.3390/gels10050324

**Published:** 2024-05-09

**Authors:** Wenjing Wang, Yadi Wang, Junhong Lü, Xueling Li

**Affiliations:** 1 Shanghai Institute of Applied Physics, Chinese Academy of Sciences, Shanghai 201800, Chinalvjunhong@jnl.ac.cn (J.L.); 2 School of Pharmacy, Binzhou Medical University, Yantai 264003, China; 3 College of Public Health, Shanghai University of Medicine & Health Sciences, Shanghai 201318, China; 4 University of Chinese Academy of Sciences, Beijing 100049, China

**Keywords:** terahertz spectroscopy, hydrogelation, poly(vinyl alcohol), copper ion, hydration layer

## Abstract

Metal-coordinated hydrogels are becoming increasingly popular in the biomedical field due to their unique properties. However, the mechanism behind gel forming involving metal ions is not yet fully understood. In this work, terahertz spectroscopy was used to investigate the role of interfacial water in the gelation process of copper ion-coordinated poly(vinyl alcohol) hydrogels. The results showed that the binding of copper ions could alter the interfacial hydration dynamics of the poly(vinyl alcohol) polymers. Combined with the results of differential scanning calorimetry (DSC), we propose a possible hydration layer-mediated mechanism for the formation of cooper ion-coordinated hydrogel during the freeze–thaw cycle. These results highlight the value of terahertz spectroscopy as a sensor for studying the hydration process in hydrogels and provide an important clue for understanding the mechanism of hydrogelation in ion-coordinated hydrogels.

## 1. Introduction

**Figure 1:**
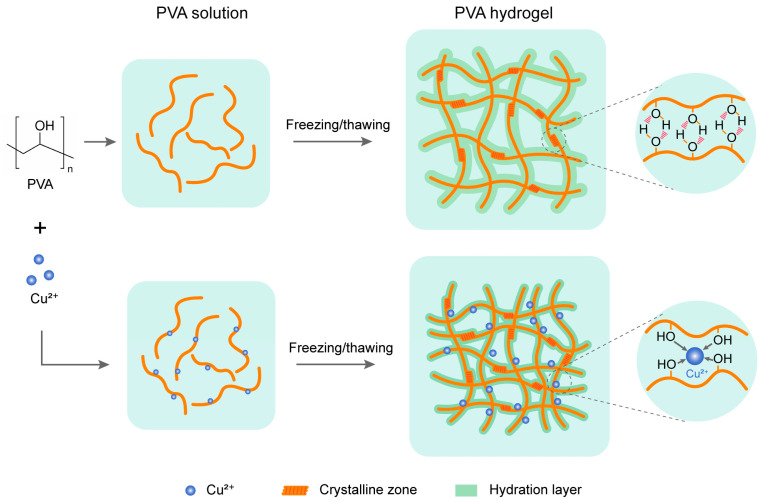
Preparation process and proposed mechanism for the PVA hydrogel formation with and without copper ions.

Polyvinyl alcohol (PVA) is a semi-crystalline polymer produced by partial or total hydrolysis of polyvinyl acetate [1,2]. Due to its excellent biocompatibility, low cost, and ease of processing, PVA has attracted increasing attention for its biomedical and pharmaceutical applications [3,4,5]. It is generally recognized that aqueous PVA solutions can be cross-linked to form a hydrogel via crystalline zones in a repeated freeze–thaw cycle [1]. During the freeze–thaw process, intermolecular hydrogen bonding could be formed by hydroxyl groups close to each other on the PVA molecular chain, leading to the formation of a crystalline zone. The crystalline area plays the role of cross-linking points for the formation of a complete gel network. The polymer crystallinites induced by freezing act as physical cross-linking points between the PVA chains and determine the structural stability and physico-chemical properties of the hydrogel (Figure 1). A large number of hydroxyl groups contained in the polymer can form a hydrogen bonding network with a tetrahedral structure and picosecond-scale dynamics [1,3]. Recent studies have shown that metal ions can significantly enhance the physical and mechanical properties of hydrogels [2,5,6,7,8]. For example, Wang et al. used PVA as the main raw material in combination with acrylic acid (AA) and metal ions to prepare a tri-crosslinked network of antimicrobial hydrogels with good mechanical properties [9]. Gao et al. prepared PVA/PAA/Fe^3+^-GaIn hydrogel with certain antimicrobial properties and a good mechanical response [10]. In these works, various methods, including FTIR, UV–vis spectra [11,12,13,14] and EDS, XRD, XPS [15,16,17,18,19] and NMR [20], were used to study the interaction between metal ions and polymers. While valuable information on the molecular structure and the ion-induced changes in the polymers that occur during the formation of hydrogels has been obtained, the role and the involvement mechanism of metal ions in hydrogel formation is not fully understood. 

Terahertz spectroscopy is a sensitive technique for sensing the dynamics of the hydrogen bonding network of water molecules in hydrogels [21,22,23]. In the terahertz region, there are multiple modes of activity of water molecules, with a dominant mediated electrical relaxation mode [23]. A well-known model used to characterize the dielectric relaxation of pure water in the terahertz region is the double Debye relaxation model [10]. It has been proposed that there are two distinct relaxation processes for water molecules in aqueous systems: a slow one (τ_1_) and a fast one (τ_2_). The slow part is called the Debye time, which results from the cooperative recombination of the hydrogen bond network. The fast part comes from less hydrogen-bonded water molecules, but its origin is unknown. Since water is responsible for the structure and properties of a hydrogel, such as stability and swelling rate, the role and kinetics of water in hydrogels has significant implications for their functional properties [24,25]. In general, the bound water and its ratio to the free or bulk water are essential for the formation of hydrogels, which are largely determined by the way the polymer network interacts with water. Several studies on the role of water and its dynamics in hydrogels have been evaluated using terahertz spectroscopy. For example, Zapanta et al. have used terahertz time-domain spectroscopy (THz-TDS) to differentiate the two microstructures of agarose hydrogels and found that the foil microstructure has a higher hydration number than the mesh microstructure [26]. Rossi et al. have used vibrational spectroscopy to study the hydrogen bonding network within cyclodextrin sponges [27]. Hoshina et al. used terahertz spectroscopy to study the structure and dynamics of bound water in poly(ethylene vinyl alcohol) copolymers [22]; Yan et al. investigated the dynamics of water molecules in polyacrylamide hydrogels [23]. Zhou et al. investigated the glucose-induced change in the hydration state of smart hydrogels by using terahertz attenuated total reflectance (THz-ATR) spectroscopy [28]. Although these previous works suggest that terahertz spectroscopy is a powerful tool to study the hydration water in hydrogels, the influence of different conditions, e.g., metal ions, on water dynamics in hydrogels remain unexplored [29]. 

In this study, we sought to investigate whether the hydration layer of PVA is mediated by the effect of copper ions on hydrogel formation. To this end, terahertz spectroscopy and differential scanning calorimetry (DSC) [30] were used to investigate the hydration dynamics of PVA solution and PVA hydrogels with and without the addition of copper ions. The spectral parameters, including refractive index and absorption coefficient, of these samples were comprehensively compared. Terahertz spectroscopy indicated that copper ions could affect the hydration dynamics of the PVA solution. DSC showed that copper ions lowered the melting temperatures (Tm) of PVA hydrogels. These results suggest a possible mechanism that the hydration layer on the PVA polymer surfaces might mediate the ion’s effects on hydrogelation and highlight the importance of terahertz spectroscopy as a sensor for the hydrogel research field. 

## 2. Results and Discussion

### 2.1. Preparation of PVA Solution and PVA Hydrogels

The different concentrations of copper ions were added and mixed to obtain the PVA solution. After that, the freeze–thaw method (freezing for 20 h and thawing for 4 h for four cycles) were used to preparing the PVA hydrogels [31], as shown in Figure 1. The prepared PVA solutions and hydrogels were used following terahertz spectroscopy and differential scanning calorimetry (DSC) experiments, respectively. Prior to the tests, the hydrogels were soaked with ultrapure water to wash off the reaction solution.

### 2.2. Tearahetz Spectra of PVA Solution at Different Concentrations 

**Figure 2:**
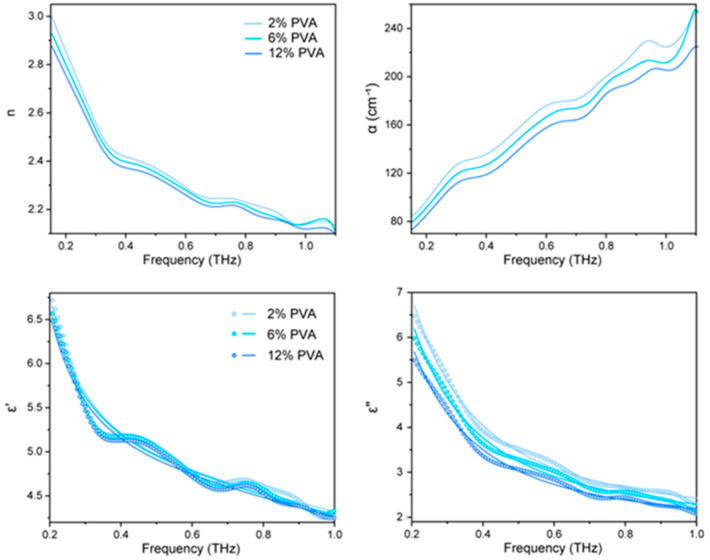
Terahertz spectra of the PVA gels formed with different concentrations.

Figure 2 displays the terahertz absorption spectra of various concentrations of PVA solutions. The results indicated that the absorption coefficient and refractive index of the aqueous solution decreased with the addition of PVA (Table 1). This reduction is further amplified with increasing PVA concentration, suggesting that the introduction of PVA affects the hydrogen bonding network of the aqueous solution, leading to changes in the vibration of the water molecule network. According to the Flory–Krigbaum theory of polymer solutions, the distribution of polymer chain segments is not uniform. Each chain segment can be viewed as a cloud of chain segments that is close to a sphere. The entire molecular chain is a loose sphere of chains made up of these segments, and the volume occupied by each polymer chain sphere is referred to as the repulsive volume. Due to a large number of hydroxyl groups distributed along the PVA molecular chain, water molecules are a suitable solvent for PVA. In the solution state, the interaction between PVA and water molecules is greater than that between the PVA molecular chain. This means that PVA in the liquid state is more likely to form hydrogen bonds with the water molecules. As a result, PVA in the solution takes the form of a loose chain sphere surrounded by water molecules. The interaction between PVA and water molecules disrupts the regular hydrogen bonding network of the original system. PVA, as a molecular chain sphere, takes the place of water molecules and further affects the water molecule network, resulting in a change in the terahertz absorption spectra. For various concentrations of PVA solution, as the concentration of PVA increases, the solution transitions from a dilute solution (2% PVA) to a critical solution (6% PVA) and then to a concentrated state (12% PVA). During the process, PVA molecules in the solution transfer from independent molecular chain spheres (each occupying an exclusive volume and not touching each other) to tightly packed spheres that do not overlap (each occupying a repulsive volume). The entire volume of the solution is made up of the repulsive volume of each molecular chain sphere. These spheres have no gaps between them, but they do not overlap. Finally, these become entangled with each other. This change not only affects the layout of the molecular chain in the network of water molecules, but also the interaction between the molecular chain and the solvent molecules. Together, these changes lead to a change in the terahertz absorption spectra. 

The calculated and fitted dielectric function of the samples is presented in terms of their real and imaginary parts. It is observed that the dielectric function exhibits a linear decrease as the PVA concentration increases, consistent with the decreasing trend of the total water molecular weight in the system. The relaxation strength of the total detected water molecules (ε_1_ + ε_2_) also decreases with increasing PVA concentration due to the decrease in the amount of total water molecules in the system. In this case, the intensity of slow relaxation water, ε_1_, decreases significantly while the intensity of fast relaxation water, ε_2_, remains unchanged. Additionally, the proportion of slow relaxation water (ε_1_/(ε_1_ + ε_2_)) decreases while the proportion of fast relaxation water (ε_2_/(ε_1_ + ε_2_)) increases. The reason for the decrease in total water molecules in the system is due to the increase in PVA. PVA replaces the original water molecules in the position, which disrupts the hydrogen bonding network structure of water molecules. As a result, the intensity of the slow relaxation water is significantly reduced. The PVA molecule chain contains numerous hydroxyl groups. In an aqueous solution, these groups preferentially form intermolecular hydrogen bonds with the water molecules, causing the PVA molecule chain to have a loose chain ball form. Therefore, the water molecules that form hydrogen bonds with PVA will be bound in the internal cavity of the PVA chain ball and the surrounding area. This results in fast relaxation water, which compensates to some extent for the reduction in the intensity of fast relaxation water due to the decrease in water molecules. As a result, the strength of this part of the water relaxation has not been significantly reduced. Although the speed of the water in the original system has decreased, the introduction of solute molecules has created new fast water, resulting in the proportion of slow water and an increase in the proportion of fast water. The binding of the PVA molecular chain sphere and water molecules results in the outer layer of water molecules wrapping around a layer of water molecules in the new chain sphere, causing an increase in volume. The increase in volume is greater than the volume of these water molecules; the volume was originally occupied by these water molecules, resulting in a certain degree of extrusion of water molecules in the bulk phase. The addition of PVA results in a more compact hydrogen bonding network, leading to a shorter relaxation time (τ_1_) of the slow relaxation water. The fast water formed under this bonding effect differs from that of the fast water in the pure water system, resulting in a different relaxation time (τ_2_). As mentioned above, the F-K dilute solution theory suggests that the distribution of polymer chains in the solution differs when transitioning from a dilute solution to a concentrated solution. This difference results in a more severe extrusion of water molecule networks in the bulk phase as the concentration of PVA increases, causing τ_1_ to decrease further. Simultaneously, the exclusion volume occupied by a single polymer chain remains constant during the transition from a dilute to a concentrated solution. As a result, the structure of the water molecules bound to the molecular chain does not change significantly with an increasing PVA concentration. Therefore, the relaxation time (τ_2_) does not change significantly with an increasing PVA concentration.

### 2.3. The Addition of Copper Ions to PVA Solutions Changes the Terahertz Dynamics

**Figure 3:**
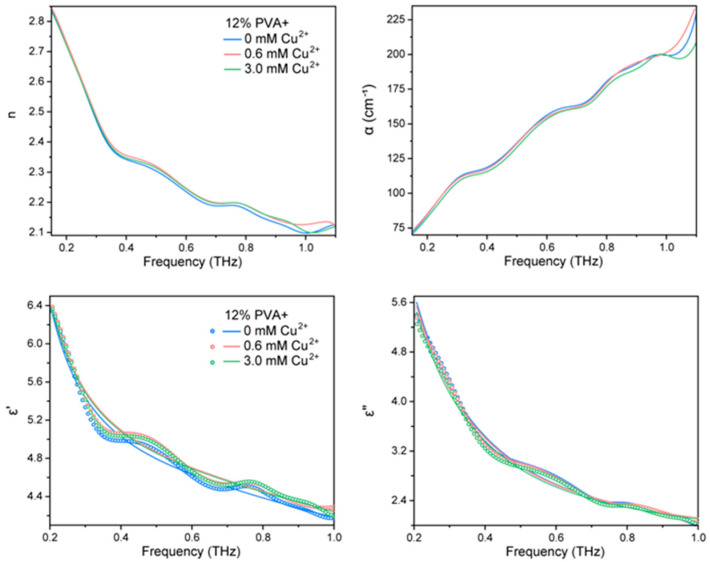
Terahertz absorption spectra of mixed solutions of 12% PVA with different concentrations of copper ions.

Figure 3 shows the terahertz absorption spectra obtained when 0.3 mM copper ions were added to the system. It is evident that the absorption coefficients and refractive indices of different concentrations of PVA did not change significantly after the addition of copper ions compared to the pure solution. The addition of copper ions is thought to have caused some damage to the network structure of the water molecules in the system. However, the amount of copper ions added was relatively small, resulting in minimal damage.

To investigate the effect of copper ions on the relaxation motion of water molecules, terahertz spectroscopy was performed on solutions with varying concentrations of copper ions added to 12% PVA concentration. Figure 3 shows the terahertz absorption spectra obtained, indicating a slight change in the absorption coefficient and refractive index with increasing copper ion concentration. To analyze the terahertz spectra, the real and imaginary parts of the dielectric function were calculated and fitted with the Debye model to obtain parameters characterizing the relaxation information of the water molecules (Table 2). The dielectric function plots show slight increases or decreases in the real and imaginary curves of the solutions with different concentrations of copper ions. It can be assumed that the addition of copper ions to the PVA solution affects the structure of the water molecules in the system and their state of motion. After the addition of copper ions, the relaxation information (ε_1_ + ε_2_) of the total water molecules in the system is reduced compared to that of the pure solution. As the copper ion concentration increases, the intensity of the slow relaxation water ε_1_ is slightly decreased, while the intensity of the fast relaxation water ε_2_ is slightly increased. The proportion of both slow relaxation water (ε_1_/(ε_1_ + ε_2_)) and fast relaxation water (ε_2_/(ε_1_ + ε_2_)) is slightly reduced. At the same time, the relaxation time of the slow water (τ_1_) decreases slightly, while the relaxation time of the fast water (τ_2_) fluctuates but in general remains almost unchanged. These results show that the addition of copper ions to PVA affects the structure and relaxation motion of water molecules in the system. Due to the interaction between the copper ions and the PVA molecular chain, and the ligand binding to the electron-rich groups on it, this behavior changes the structure and number of water molecules bound around the molecular chain. The water molecules in the bulk phase also change so that the ratio of slow water to fast water in the system and the relaxation time change accordingly.

### 2.4. Tearahetz Spectra of PVA Solution at Different Concentrations 

**Figure 4:**
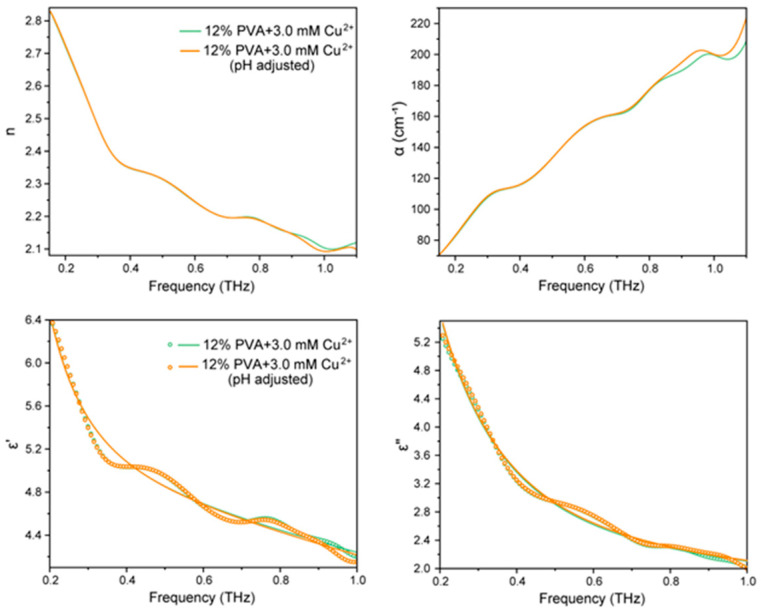
The influence of pH values on the terahertz dynamics in Cu-PVA hydrogel.

It has been reported that pH increases the electronegativity of the oxygen atom on the hydroxyl group, which enhances the coordination between the hydroxyl group and the copper ion, thus increasing the cross-linking between the chains of the PVA molecules to form a gel. To further investigate the effect of the addition of copper ions on the structure and motion state of the water molecules in the system after adjusting the pH, terahertz spectroscopy was performed on a mixed solution with the addition of 3 nM copper ions at a concentration of 12% PVA. As shown in Figure 4 and Table 3, the change in absorption coefficient and refractive index caused by the change in pH is very small. Therefore, it is assumed that the act of adjusting the pH has some effect on the structure and movement of the water molecules in the system. The relaxation information (ε_1_ + ε_2_) of the total water molecules in the system is increased compared to that of the pure solution; in this process, the intensity ε_1_ and the proportion (ε_1_/(ε_1_ + ε_2_)) of the slow relaxing water are slightly increased while the intensity ε_2_ and the proportion (ε_2_/(ε_1_ + ε_2_)) of the fast relaxing water are slightly decreased, and at the same time, the relaxation time (τ_1_) of both the slow relaxing water and the fast relaxing water (τ_2_) is slightly increased. From the analysis of the UV absorption results we already know that adjusting the pH value is more conducive to the ligand binding between the electron-rich functional groups on the PVA and the copper ions, under which the functional groups on the PVA and the copper ions will form ligand binds with stronger interactions. This process releases into the bulk phase some of the water molecules that were originally bound around the molecules but not detected due to the tight binding, leading to a change in the relaxation strengths and ratios of the two types of water detected; at the same time, this new binding makes the molecular chains tighter together, which occupies less space, leaving more space for the water molecules, resulting in a more unrestricted activity of the water molecules and thus an increase in the relaxation time of the water molecules. It can be concluded that adjusting the pH affects the network structure and movement of water molecules in the system by influencing the interaction between PVA and copper ions. As the interaction between PVA and copper ions is enhanced after pH adjustment, the coordination mode is changed; this new coordination bond releases some water molecules that are too tightly bound around the molecule to be detected into the bulk phase, and the PVA molecular chains become more tightly bound to each other, which also releases more space and makes the bonded water around it as well as the bulk water relatively loose, thus causing a series of changes as mentioned above.

### 2.5. Thermodynamic Properties of Copper Ion–PVA Hydrogel

**Figure 5:**
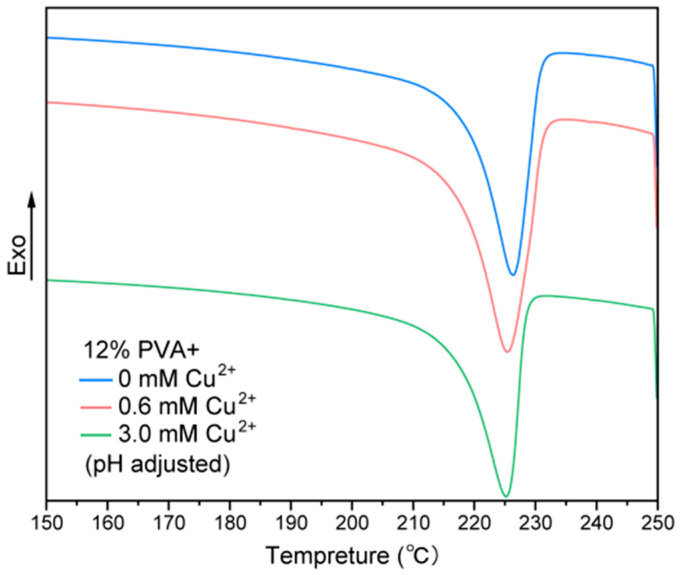
DSC curves of the copper ion gradient PVA hydrogel sections, demonstrating their melting temperatures (Tm). The Tm values of the hydrogel sections slightly decrease with the increasing copper ion concentration stiffness from 0 mM to 3.0 mM, indicating the gradually decreasing crystallinity and possibly the folding length of the crystallites.

In order to investigate the crystallite formation characteristics between the PVA chains in copper ion-containing hydrogel, the melting temperature (Tm) was determined using differential scanning calorimetry (DSC). The calculation of crystallinity requires the melting enthalpy of PVA with 100% crystallization, but we have not found the exact value. According to the crystallinity calculation formula, xc = (∆H/∆H0) × 100% (∆H is the molar melting enthalpy of the sample and ∆H0 is the molar melting enthalpy of the sample at complete crystallization), ∆H is used here to compare the relative size of the sample’s crystallinity. From the thermal degradation behavior of the prepared samples, 250 °C was chosen as the highest heating temperature for the thermal analysis of the gels. The experimental results obtained from the DSC experiments on the gels were plotted and analyzed using the origin, and the results are shown in Figure 5. The sharp peaks around 220–230 °C in the DSC curves correspond to the Tm of the crystallites in the PVA hydrogel. The Tm values of the hydrogel sections decrease slightly with increasing copper ion concentration from 0 mM to 3.0 mM, indicating the gradually decreasing crystallinity and possibly the folding length of the crystallites. These results further support the idea that the effect of copper ions on the hydrogel formation may be mainly due to the ionic binding-induced hydration layer change in the PVA polymer.

## 3. Conclusions

In this study, we used terahertz spectroscopy as a sensor to detect the hydration dynamics of the PVA solution under different copper ion concentrations. The spectral parameters, including refractive index and absorption coefficient, of these samples were comprehensively compared. DSC was also used to investigate the effect of copper ions on the Tm of hydrogels. We found that the copper ions could affect the hydration states of PVA polymers in a concentration-dependent manner. It is generally recognized that the crystalline zone plays an important role in the hydration formation of PVA solution during a freeze–thaw cycle. However, the underlying mechanism is not fully understood. In particular, the role of metal ions in the changes in hydrogel formation and its properties remain elusive. Considering the fact that the absorption coefficient decreased with the increasing PVA concentration, we proposed that the hydration layer, not only the crystalline zone, might play an important role in the hydration formation. The changes in the hydration layer induced by the binding of copper ions to PVA polymers not only contribute to the hydrogel formation, but also relate to the hydrogel properties. The copper ion-induced reduction in the hydration layer makes gel formation more difficult, but the formed hydrogel has higher mechanical properties due to the tighter packing of polymer molecules (Figure 1). These findings demonstrate the value of terahertz spectroscopy for hydrogel research, providing terahertz dynamic insight into the mechanism of hydrogelation and the effect of metal ions on the hydrogel formation. 

## 4. Materials and Methods

### 4.1. Materials

The PVA used in this study had a molecular weight of 124–146 kg mol^−1^ and was 98–99% hydrolyzed. It was purchased from Shanghai Macklin Biochemical Technology Co. CuCl_2_·2H_2_O, NaOH and phosphate-buffered saline (PBS) were purchased from Hunan BKMAM BIOTECHNOLOGY Co (Shanghai, China).

### 4.2. Preparation of PVA-Cu Hydrogels

The PVA solution (12% *w*/*v*) was prepared by dissolving PVA in Millipore water at 90 °C with constant stirring overnight. The solution was sealed throughout the process to prevent water evaporation. Next, 1 M CuCl_2_ water solution was added in varying molar ratios. Finally, NaOH solution was added to the mixture to adjust the pH. The hydrogel prefilled Petri dishes were stored overnight at −18 °C and underwent four freeze–thaw cycles.

### 4.3. DSC Measurement [30]

The gel, which had been freeze-dried, was cut into 5–10 mg pieces using scissors. These pieces were then placed flat on the bottom of an aluminum crucible and measured according to the set procedure. The melting temperature (Tm) was measured by eliminating the thermal history and then raising the temperature again. A heating rate of 10 °C/min was used for both heating and cooling under a N_2_ atmosphere of 50 mL/min. The temperature was first programmed to increase from 30 °C to 235 °C, held at 235 °C for 2 min to eliminate the thermal history, then cooled down to 30 °C, and finally increased to 250 °C to measure the temperature transition during the warming process.

### 4.4. Terahertz Spectroscopy Measurement

The samples were measured by means of THz time-domain spectroscopy (THz-TDS) in the frequency range 0.1–4.0 THz. A titanium gemstone femtosecond laser was used as the light source with a pulsed laser wavelength of 800 nm, a pulse width of 35 fs, a repetition frequency of 1000 Hz, and a signal-to-noise ratio of about 70 dB. Terahertz waves were generated and detected in the system using a photoconductive antenna. THz-TDS is a coherent detection technique that can simultaneously obtain the amplitude and phase information of the terahertz pulse. By analyzing the waveform in both the time domain and frequency domain, parameters such as refractive index and absorption coefficient can be determined. To prevent water vapor absorption, the entire terahertz experimental setup was placed in a laboratory with controlled temperature and humidity. The temperature was controlled at 21.0 ± 0.5 °C and the humidity was controlled within 1%. A cuvette made of fused silica with a thickness of a sub-millimeter order of magnitude was selected. Prior to measurement, the cuvette’s sample clip was fixed in the optical path, ensuring that the light wave passed through the same position of the cuvette for each measurement. An empty cuvette was selected as the reference signal during the measurement. Each set of samples was measured at least three times.

### 4.5. Data Analysis

The time-domain signal, which includes the amplitude and phase of the terahertz electromagnetic wave, is analyzed using the principle of coherent detection. The analysis allows for the calculation of the sample’s complex refractive index (expressed by the real part of the refractive index and the absorption coefficient) and the complex permittivity (including the real part and the imaginary parts of the permittivity) in the terahertz wavelength band, which are calculated based on the analyzed signal.

At the end of the measurement, a fast Fourier transform is performed on the terahertz time-domain pulse signal to obtain the power and phase of the terahertz pulse in the frequency domain. This information is then used to calculate the sample. Due to the weak surface reflection of the sample. The refractive index n_s_(ν), absorption coefficient α_s_(ν) and the extinction coefficient *k_s_*(ν) of the sample were obtained using the following equation:

(1) $$n_{s}(\nu )=n_{r}(\nu )+\left[\Phi_{r}(\nu )-\Phi_{s}(\nu )\right]\times \frac{C}{2\pi \nu d}$$

(2) $$\alpha_{s}(\nu )=\frac{2}{d}\cdot \ln\left[\frac{4n_{r}(\nu )n_{s}(\nu )}{\frac{I_{s}(\nu )}{I_{r}(\nu )}\cdot {\left(n_{r}(\nu )+n_{s}(\nu )\right)}^{2}}\right]$$

(3) $$k_{s}(\nu )=\frac{\alpha_{s}(\nu )}{2\pi v}$$

where d is the distance that light passes through the sample, i.e., the thickness of the cuvette, I_r_(ν) and I_s_(ν) are the intensities of the empty cuvette and the sample solution as a reference, respectively, n_r_(ν) is the refractive index of the reference, and Φ_s_(ν) and Φ_s_(ν) are the reference phase and the sample phase, respectively.

The real part ε′ and the imaginary part ε″ of the sample dielectric constant can be calculated from the following equation:

(4) $$\varepsilon^{\prime }=n_{s}^{2}(\nu )-{k_{s}}^{2}$$

(5) $$\varepsilon^{\prime \prime }=2n_{s}(\nu )\cdot k_{s}$$

The Debye model was used to fit ε′ and ε″ in order to obtain information on water molecule relaxation in the sample. This includes the total amount of total water molecules in the system (ε_∞_), the amount of water in the bulk phase (ε_1_), the amount of water at the interface (ε_2_), the slow relaxation time of water molecules (τ_1_), the fast relaxation time of water molecules (τ_2_), as well as the real part (εf′) and the imaginary part (εf″) of the dielectric constant.

## Figures and Tables

**Table 1:** Terahertz absorption coefficient α(ν) and the refractive index n(ν) of PVA with different concentrations.

	ε_∞_	ε_1_	ε_2_	ε_1_ + ε_2_	ε_1_/(ε_1_ + ε_2_)	ε_2_/(ε_1_ + ε_2_)	τ_1_	τ_2_
2% PVA	2.279	21.271	2.337	23.608	90.10%	9.90%	2.461	0.0816
6% PVA	2.416	19.798	2.213	22.011	89.95%	10.05%	2.423	0.0845
12% PVA	2.603	17.178	2.016	19.194	89.50%	10.50%	2.289	0.0944

**Table 2:** Terahertz absorption coefficient α(ν) and the refractive index n(ν) of PVA with different copper ion concentrations.

12% PVA	ε_∞_	ε_1_	ε_2_	ε_1_ + ε_2_	ε_1_ /(ε_1_ + ε_2_)	ε_2_ /(ε_1_ + ε_2_)	τ_1_	τ_2_
+0 mM Cu^2+^	2.668	18.687	1.991	20.678	90.37%	9.63%	2.441	0.099
+0.6 mM Cu^2+^	2.663	18.170	2.059	20.229	89.82%	10.18%	2.394	0.097
+3.0 mM Cu^2+^	2.622	17.738	2.090	19.828	89.46%	10.54%	2.377	0.095

**Table 3:** Terahertz absorption coefficient α(ν) and the refractive index n(ν) of PVA–copper ion solution at different pH levels.

12% PVA	ε_∞_	ε_1_	ε_2_	ε_1_ + ε_2_	ε_1_ /(ε_1_ + ε_2_)	ε_2_ /(ε_1_ + ε_2_)	τ_1_	τ_2_
pH 4.0	2.622	17.738	2.090	19.828	89.46%	10.54%	2.377	0.095
pH 7.5	2.683	18.162	2.055	20.217	89.84%	10.16%	2.437	0.102

## Data Availability

Data are contained within this manuscript.
